# Clinical manifestations of dual-gene variants involving *ABCA4* in retinal dystrophies

**DOI:** 10.1186/s12886-025-04048-1

**Published:** 2025-04-23

**Authors:** Lasse Wolfram, David A. Merle, Laura Kühlewein, Milda Reith, Melanie Kempf, Krunoslav Stingl, Tobias Haack, Pascale Mazzola, Karin Poths, Nicole Weisschuh, Bernd Wissinger, Susanne Kohl, Katarina Stingl

**Affiliations:** 1 Department for Ophthalmology, University Eye Clinic, Eberhard Karls University of Tübingen, 72076 Tübingen, Germany; 2 Department for Ophthalmology, Institute for Ophthalmic Research, Eberhard Karls University of Tübingen, 72076 Tübingen, Germany; 3 Center for Rare Eye Diseases, Eberhard Karls University of Tübingen, 72076 Tübingen, Germany; 4 Institute of Medical Genetics and Applied Genomics, Eberhard Karls University of Tübingen, 72076 Tübingen, Germany

**Keywords:** Inherited retinal diseases, Retinal dystrophies, Dual-gene variants, Genotype-phenotype correlations, ABCA4, CACNA1F, IMPG1, HK1, MYO7A

## Abstract

**Background:**

This study investigates the clinical manifestations of inherited retinal diseases (IRD) associated with dual-gene variant constellations involving biallelic *ABCA4* variants.

**Methods:**

We assess four cases for their unique phenotypic outcomes due to biallelic *ABCA4* variants and additional genotypes in *CACNA1F*, *IMPG1*, *HK1* and *MYO7A*, respectively.

**Results:**

This study investigates the phenotypic impact of dual-gene variants, including biallelic *ABCA4* variants and additional retinal gene variants in *CACNA1F*, *IMPG1*, *HK1* and *MYO7A*. In MST465-II:1, the *ABCA4*-*CACNA1F* constellation led to progressive macular atrophy and night blindness, with nystagmus linked to *CACNA1F*. In MST448-II:1, *ABCA4* variants primarily contributed to a macular dystrophy, while the *IMPG1* variant had no obvious impact, suggesting it may be a benign polymorphism. In SRP1400-II:1, a *de novo HK1* variant caused retinitis pigmentosa (RP)-like retinal degeneration and intellectual disability and in USHI105-II:1, *MYO7A* variants primarily resulted in an Usher syndrome 1 phenotype. In both latter cases, *ABCA4* variants play a more subtle role. These findings illustrate the importance of critical phenotype and genotype assessment and how complex interactions between *ABCA4* and other genetic variants can configure the phenotype, making it challenging to distinguish the contributions of each gene.

**Conclusions:**

This study underscores the importance of advanced diagnostic tools and careful genotype evaluation to accurately identify and understand potential complex genetic interactions in IRDs. The observed phenotypes enhance our understanding of how these genes contribute to human retinal function and dysfunction. Furthermore, these insights can impact clinical decision-making, as patients with dual-gene variant constellations might experience questionable benefit from potential future gene therapies. Thus, careful patient selection and complete genotype and phenotype assessment before treatment is essential to manage potential risks and costs effectively.

## Background

Inherited retinal diseases (IRDs) encompass a diverse group of genetic conditions that impair retinal structure and function, leading to vision loss and often resulting in profound visual impairment or blindness. IRDs exhibit considerable clinical and genetic heterogeneity with more than 250 associated genes identified to date [1, 2]. Many of these conditions are generalized retinal disorders and can be classified into rod-cone-, cone-(rod-) and macular dystrophies based on the type of photoreceptor affected and the location within the retina.

Entities discussed in this manuscript include *ABCA4*-associated IRDs, such as cone-rod dystrophy (CORD3), Stargardt disease type 1 (STGD1) and retinitis pigmentosa (RP; RP19), as well as *CACNA1F*-associated incomplete congenital stationary night blindness (iCSNB; CSNB2A), *IMPG1*-associated vitelliform macular dystrophy (VMD4) and RP (RP91), *HK1*-associated RP (RP79) and *MYO7A*-associated Usher syndrome (USH1B). Despite advances in understanding the genetic basis of IRDs, many challenges remain in elucidating their complex pathophysiology, developing effective treatments and providing personalized management strategies. Genotype-phenotype correlation often is not straightforward and, in many cases, not well understood, as exogeneous and further modifying genetic factors may play an important role in the phenotype expression. The intricate interplay of genotypic and phenotypic variation presents challenges in both diagnosis and comprehension. This complexity underscores the importance of detailed genetic and phenotypic profiling to ensure accurate diagnosis and effective management of IRDs [3].

Recent progress in sequencing technologies enables the screening of the entire exome or even genome, which contributes to a higher rate of conclusive results for patients with IRDs [4, 5]. These possibilities to analyze all known IRD-associated genes, including non-syndromic and syndromic, in a simultaneous workup, however, can reveal the presence of pathogenic variants in two or more distinct genes associated with IRDs, which would not necessarily be found in single gene sequencing or limited panel testing. These multigene compound genotypes can lead to an altered and potentially more severe phenotype when compared to individuals carrying variants in only a single gene [6].

*ABCA4* is one of the most frequently mutated genes associated with IRDs [7] and STGD1 is the most common inherited macular degeneration in working-aged individuals. While a global prevalence of 1 in 10,000 is widely cited in the literature, more recent studies suggest a lower prevalence of approximately 1 in 20,000 [8, 9]. The *ABCA4* gene encodes an ATP-binding cassette (ABC) superfamily transmembrane protein crucial for the transport of retinoids in photoreceptor cells. Pathogenic variants in *ABCA4* potentially disrupt this process, leading to the accumulation of toxic compounds in the retinal pigment epithelium, which subsequently causes photoreceptor degeneration [10]. *ABCA4* variants were initially associated with STGD1, a common inherited macular dystrophy, but can also cause other IRD forms, such as cone-(rod) dystrophy (CORD3) and RP (RP19) [8, 11]. *ABCA4*-related disorders follow an autosomal recessive inheritance pattern, requiring mutations on both alleles for the disease to manifest. *ABCA4* variants are highly prevalent in the general population, with carriers of heterozygous mutations found in approximately 2–5% of the individuals [12]. This high carrier frequency contributes to the relatively common occurrence of *ABCA4*-related disorders when compared with other IRDs. Recent studies have identified over 2,000 variants in the *ABCA4* gene, including missense, nonsense, frameshift, splicing and structural variants [13]. Yet it is long known that severity of *ABCA4*-related disease depends on the genotype and efforts have been undertaken to categorize *ABCA4* variants in benign, hypomorphic, moderate/mild and severe [7, 14]. The complexity of this gene and the variability in clinical presentation make interpretation of results from genetic testing and accurate diagnosis and prognosis sometimes challenging, yet essential, especially as targeted therapies are being developed [15].

So far, only few cases with pathogenic variants in two genes involving *ABCA4* have been previously documented. Huynh and co-workers (2014) reported a case of a female patient with STGD1 and complete congenital stationary night blindness (CSNB1B), carrying biallelic *ABCA4* variants (p.(Leu1201Arg) and p.(Arg2077Gly)) along with biallelic *GRM6* gene variants (c.50_64del and c.1835_1837del) [16]. Similarly, Lee and colleagues (2016) described two female patients with STGD1 who were also carriers of ocular albinism [17]. These patients exhibited *ABCA4*-associated changes such as bull’s eye maculopathy and retinal mosaic patterns characteristic of Nettleship-Falls type ocular albinism (OA1). They carried biallelic *ABCA4* variants (p.(Leu541Pro) and p.(Gly1961Glu)) and a heterozygous *GPR143* gene variant (p.(Tyr257Cys)). Hayashi and co-workers (2020) reported two additional cases of patients with overlapping phenotypes of cone-rod dystrophy (CORD3) and Nougaret-type congenital stationary night blindness (CSNBAD3), carrying biallelic *ABCA4* variants (p.(Gln185Ter) and c.1760 + 2T > G) alongside a heterozygous dominant *GNAT1* variant (p.(Gly38Asp)) [18]. Stevanovic and colleagues (2023) presented a case with a family history of *PRPH2*-associated pattern dystrophy (MDPT1) but more extensive retinal disruption and functional impairment compared to their asymptomatic parent with only limited macular abnormalities. Genetic testing revealed the familial pathogenic *PRPH2* variant and two pathogenic biallelic *ABCA4* variants [19]. These reports highlight the complexity and variability in phenotypic expression among individuals with overall rare dual-gene variant constellations.

In this study, we explored genotype-phenotype correlations in four individuals from four families with *ABCA4*-related dual-gene variant constellations as well as additional family members with related genotypes. This work analyzes the potential impact of individual genetic variants and for at least two cases their complex contribution on disease manifestation and progression. The subsequent discussion on implications of dual-gene variant constellations in the context of genetic counseling and potential future therapeutic interventions for IRDs highlights the importance of comprehensive molecular genetic testing including detailed family segregation analysis in the process of defining accurate clinical and genetic diagnosis.

## Methods

### Patient selection

This retrospective study analyzed genetic and clinical data from four IRD patients with an *ABCA4*-related dual-gene variant constellation as well as two affected siblings (MST465-II:2, MST465-II:3) and one unaffected mother (USHI105-I:2), each carrying only one of the identified genotypes. All seven individuals presented at the University Eye Hospital in Tübingen (Germany).

### Ophthalmological examination

Clinical examination included best-corrected visual acuity (BCVA), slit-lamp biomicroscopy and dilated fundoscopy, pseudocolor fundus photography (PCFP; California P200DTx, Optos, Dunfermline, UK), fundus autofluorescence imaging (FAF; California P200DTx, Optos, Dunfermline, UK), optical coherence tomography (OCT; Spectralis, Heidelberg Engineering, Heidelberg, Germany), 90° semi-automated kinetic perimetry (Goldmann visual field, GVF; Octopus 900, Haag-Streit Diagnostics, Köniz, Switzerland), full-field electroretinography (ffERG) measuring dark-adapted (DA) and light-adapted (LA) responses following the International Society for Clinical Electrophysiology of Vision (ISCEV) standard and full-field stimulus threshold testing (FST with 0 dB set to 0.01 cd.s/m^2^; Espion 2 and Espion 3, Diagnosys, Lowell, MA, USA) measuring dark adapted thresholds using blue and red lights.

### Genetic testing

Initial diagnostic genetic workup of the index patients included virtual non-syndromic and syndromic IRD gene panel testing based on whole genome sequencing at the Institute of Medical Genetics and Applied Genomics, University of Tübingen, Tübingen, Germany [5], conducted either on the patient alone or as part of a trio sequencing approach. Trio sequencing refers to the genetic analysis of an affected individual along with both biological parents. This approach helps determine the inheritance pattern, identify *de novo* variants and improve variant interpretation by distinguishing between inherited and spontaneous mutations [20]. Segregation analysis was done either in a diagnostic or research setting by polymerase chain reaction (PCR) amplification of the variant-carrying exons and subsequent Sanger sequencing. Variant assessment followed the American College of Medical Genetics and Genomics (ACMG) guidelines for pathogenicity classification. In addition, the findings of Cornelis et al., 2023 for severity evaluation of *ABCA4* variants were considered for their contribution to the phenotype (Table 1) [7, 21].

**Table 1:** Patients’ genotypes, ACMG variant classification of all variants and *ABCA4* variant severity category according to Cornelis et al. 2023 [7]

Patient / Family	***ABCA4*** genotype	ACMG classification	***ABCA4*** severity category*	Other IRD-related genotype	ACMG classification
**MST465**	c.1622T > C;p.Leu541Pro [22]	P	Severe	***CACNA1F***	
	c.3113 C > T;p.Ala1038Val [22]	P	Severe	c.3166dup; p.Leu1056ProfsTer11 [23]	P
	c.5313–2 A > G;p.? [5]	LP	n.a.		
**MST448**	c.181 A > G;p.Met61Val [5]	LP	n.a.	***IMPG1***	
	c.3703 A > G;p.Asn1235Asp [24]	LP	Mild/Moderate	c.151dup; p.Met51AsnfsTer29 [5]	P
**SRP1400**	c.2588G > C;p.Gly863Ala [25]	LP	Mild	***HK1***	
	c.5642 C > T;p.Ala1881Val [26]	VUS/LP	n.a.	c.1334 C > T;p.Ser445Leu [27]	LP
**USHI105**	c.4539 + 859 C > T;p.? [14]	VUS	Benign	***MYO7A***	
	c.5461-10T > C;p.? [28]	P	Severe	c.1555–8 C > G;p.? [30]	P
	c.5603 A > T;p.Asn1868Ile [29]	VUS	Mild	c.3503G > A;p.Arg1168Gln [31]	P

Footnote: *ABCA4 severity with respect to phenotypic contribution according to Cornelis et al. 2023 [7]. n.a., not available – variant has not been assessed in Cornelis et al. 2023 [7]. B, benign; VUS, variant of uncertain significance; LP, likely pathogenic; P, pathogenic

## Results

### Family MST465: *ABCA4 & CACNA1F*

The male patient MST465-II:1 was clinically diagnosed with cone-rod dystrophy. His genetic testing revealed a dual-gene variant constellation with three heterozygous variants in *ABCA4* and one hemizygous variant in *CACNA1F*. Subsequent segregation analysis confirmed biallelic status for the *ABCA4* variants: On the maternal allele the patient inherited the well-known complex allele consisting of the pathogenic missense variants c.1622T > C, p.(Leu541Pro) (severe) and c.3113 C > T, p.(Ala1038Val) (mild) in *cis* and on the paternal allele the likely pathogenic splice acceptor site variant c.5313–2 A > G, p.(?). The latter is a novel variant and unique to this family affecting the canonical acceptor site AG dinucleotide and we consider this as a severe variant like other canonical splice site variants in *ABCA4*. Additionally, the patient MST465-II:1 had a hemizygous pathogenic frameshift variant c.3166dup, p.(Leu1056ProfsTer11) in the *CACNA1F* gene for which his mother was shown to be a carrier.

**Fig. 1:**
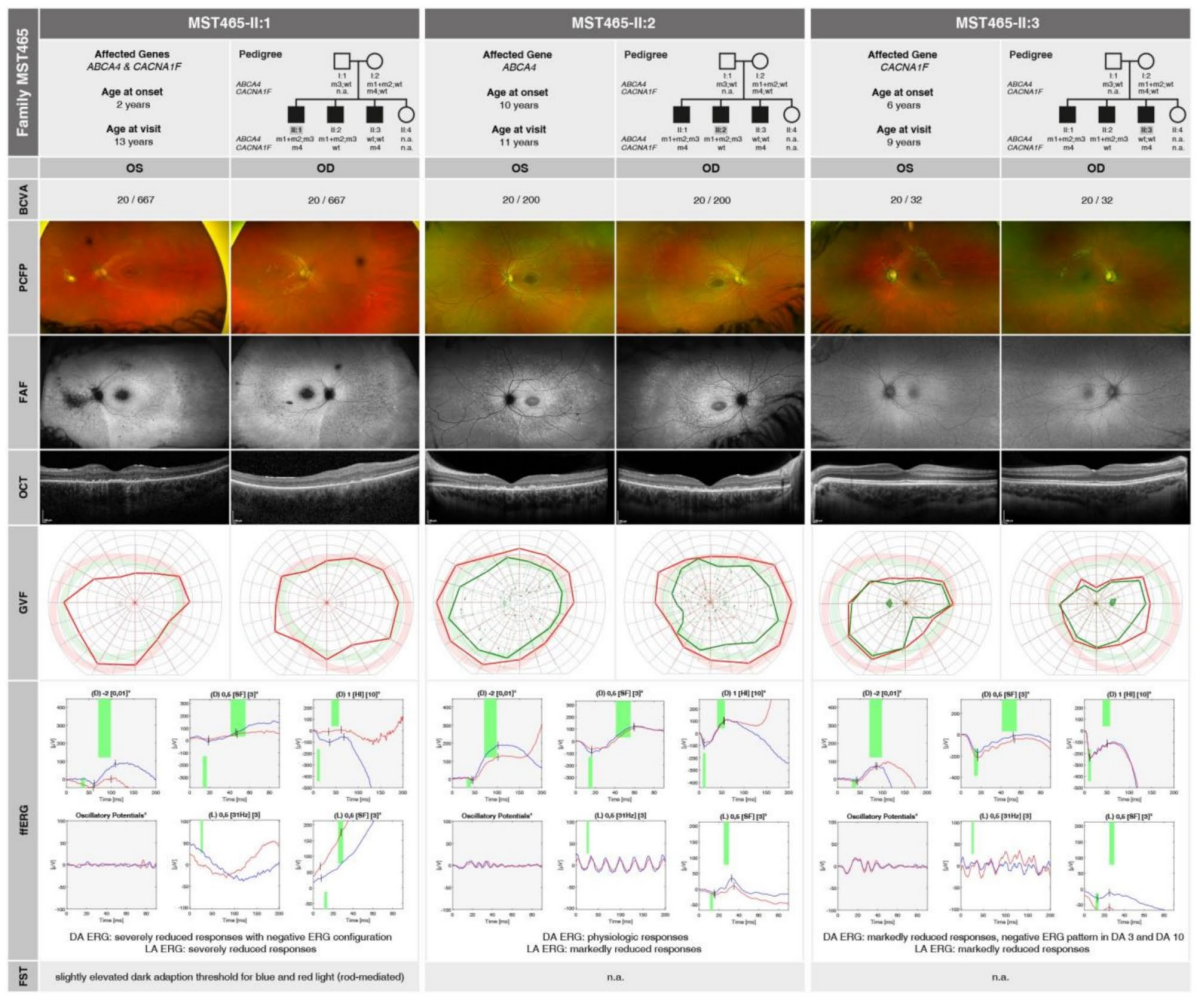
Pedigree analysis and summary of morphological and functional findings. The left panel shows **MST465-II:1** at the age of 13 years, the central panel displays **MST465-II:2** at the age of 11 years and the right panel presents **MST465-II:3** at the age of 9 years. The identified mutations are represented as follows: m1: *ABCA4* c.1622T > C, p.(Leu541Pro); m2: *ABCA4* c.3113 C > T, p.(Ala1038Val); m3: *ABCA4* c.5313–2 A > G, p.(?); m4: *CACNA1F* c.3166dup, p.(Leu1056ProfsTer11). The ffERG traces are presented with a grey background to indicate DA conditions and a white background to indicate LA conditions

MST465-II:1 initially presented to our clinic at the age of 10, reporting night blindness, photophobia and reduction in visual acuity since the age of 2 years. At the time of consultation, his BCVA was reduced to 20/250 in the right eye (-1.00 DS / -1.00 DC × 180°) and 20/400 in the left eye (0.00 DS / -1.50 DC × 160°). The anterior segment was unremarkable. Retinal examination and imaging revealed central atrophy of the outer retinal layers, along with flavimaculatus flecks typical for the *ABCA4* phenotype. FAF showed central hypoautofluorescence, accompanied by diffuse hyperautofluorescence and small, patchy hypo-autofluorescent spots in the mid-periphery. Perimetry demonstrated physiological outer borders with target III4e. At a follow-up visit at the age of 11 years, his BCVA had slightly declined to 20/320 in the right eye and remained stable at 20/400 in the left eye. Progression of atrophy and photoreceptor clumping in the central macula was observed, though GVF showed no changes. Electrophysiologic examination with ffERG revealed markedly reduced responses under DA but especially LA conditions. Despite the strongly reduced responses, the negative configuration of the mixed scotopic responses, typical for *CACNA1F* phenotypes, was present with the b-wave not exceeding the baseline. At the age of 13 years, MST465-II:1 reported further visual deterioration and occasional nystagmus. His BCVA had further decreased to 20/667 in both eyes. Retinal examination revealed continued progression of central outer retinal atrophy and increased peripheral retinal involvement, consistent with cone-rod dystrophy. ffERG revealed still severely reduced responses and FST indicated a rod-mediated, slightly elevated dark adaptation threshold for blue (-46.98 dB) and red (-23.05 dB) light. Outer borders of the visual field were still nearly normal. A summary of genetic and clinical findings is provided in Fig. 1, **MST465-II:1**.

His two years younger brother, MST465-II:2, carried the same biallelic *ABCA4* genotype as observed in MST465-II:1: c.[1622T > C;3113 C > T]; [5313–2 A > G], p.[(Leu541Pro); (Ala1038Val)]; [(?)], but not the *CACNA1F* variant. He presented to our clinic at the age of 11 years, reporting reading difficulties and photophobia. At the time of consultation, his BCVA was severely reduced to 20/200 in both eyes (OD: -0.25 DS / -1.75 DC × 20°; OS: -0.50 DS / -0.75 DC × 160°). The anterior segment examination was unremarkable. Retinal examination and imaging revealed atrophy of the outer retinal layers in the central macula with corresponding hypoautofluorescence and the presence of flavimaculatus flecks associated with diffuse hypo- and hyperautofluorescence at the posterior pole. GVF demonstrated physiological outer borders. ffERG showed borderline physiologic responses under DA conditions with a slightly smaller a-wave in the 10 DA ERG, but markedly reduced responses under LA conditions. All in all, MST465-II:2 presents as a case of STGD1, exhibiting relatively severe visual impairment already at a young age. A summary of genetic and clinical findings is provided in Fig. 1, **MST465-II:2**.

MST465-II:1’s four years younger brother, MST465-II:3, was hemizygous for the c.3166dup, p.(Leu1056ProfsTer11) variant in the *CACNA1F* gene, but did not inherit any of the *ABCA4* variants segregating in his family. He first presented to our clinic at the age of 6 years. At the time of consultation, his BCVA was reduced to 20/32 in both eyes (OD: +1.75 DS / -0.75 DC × 20°; OS: +1.25 DS / -0.50 DC × 15°) with unremarkable morphological findings from anterior and posterior segment examinations, including OCT diagnostics. During a follow-up visit at the age of 9 years, MST465-II:3 reported reading difficulties, although he managed well when seated in the front row at school. Furthermore, he experienced photophobia. At this consultation, his BCVA was stable and morphological findings, including OCT and FAF, remained unremarkable. GVF revealed near-to-normal visual field borders, likely affected by the patient’s young age and limited cooperation during the consultation. ffERG demonstrated reduced DA responses with a negative ERG pattern in both the DA 3 ERG and the DA 10 ERG, as well as markedly reduced responses under LA conditions. Overall, MST465-II:3 presents as a case of iCSNB, marked by pronounced photophobia that exceeds the degree of night blindness. He showed a comparatively mild and stable functional impairment and no pathological findings on morphological examination. A summary of genetic and clinical findings is provided in Fig. 1, **MST465-II:3**.

The youngest sibling, an 8 years younger sister, presented to our clinic at the age of 5 years asymptomatically. Given no abnormalities in thorough morphological and functional examinations and the absence of any clinical indications of IRD at the time of consultation, predictive genetic testing was not conducted. The parents did not exhibit any ophthalmological abnormalities and were carriers of the *ABCA4* and *CACNAF1* variants.

### Family MST448: *ABCA4 & IMPG1*

MST448-II:1, a male patient, was clinically diagnosed with STGD1. Genetic analysis showed that he carried two heterozygous likely pathogenic missense variants in the *ABCA4* gene, c.181 A > G, p.(Met61Val), which is novel and unique to this patient (not mentioned in Cornelis et al., 2023 [7]) and the variant c.3703 A > G, p.(Asn1235Asp) (mild to moderate), along with a heterozygous frameshift variant in the *IMPG1* gene (c.151dup, p.(Met51AsnfsTer29)) that was classified as pathogenic. Variants in *IMPG1* have been associated with autosomal recessive and autosomal dominant vitelliform macular dystrophy (VMD4) as well as autosomal dominant RP (RP91). As segregation analysis was not possible in his family, the *trans* configuration of the *ABCA4* variants could formally not be proven and it remains elusive whether this *IMPG1* variant is *de novo* or inherited from one of his parents.

**Fig. 2:**
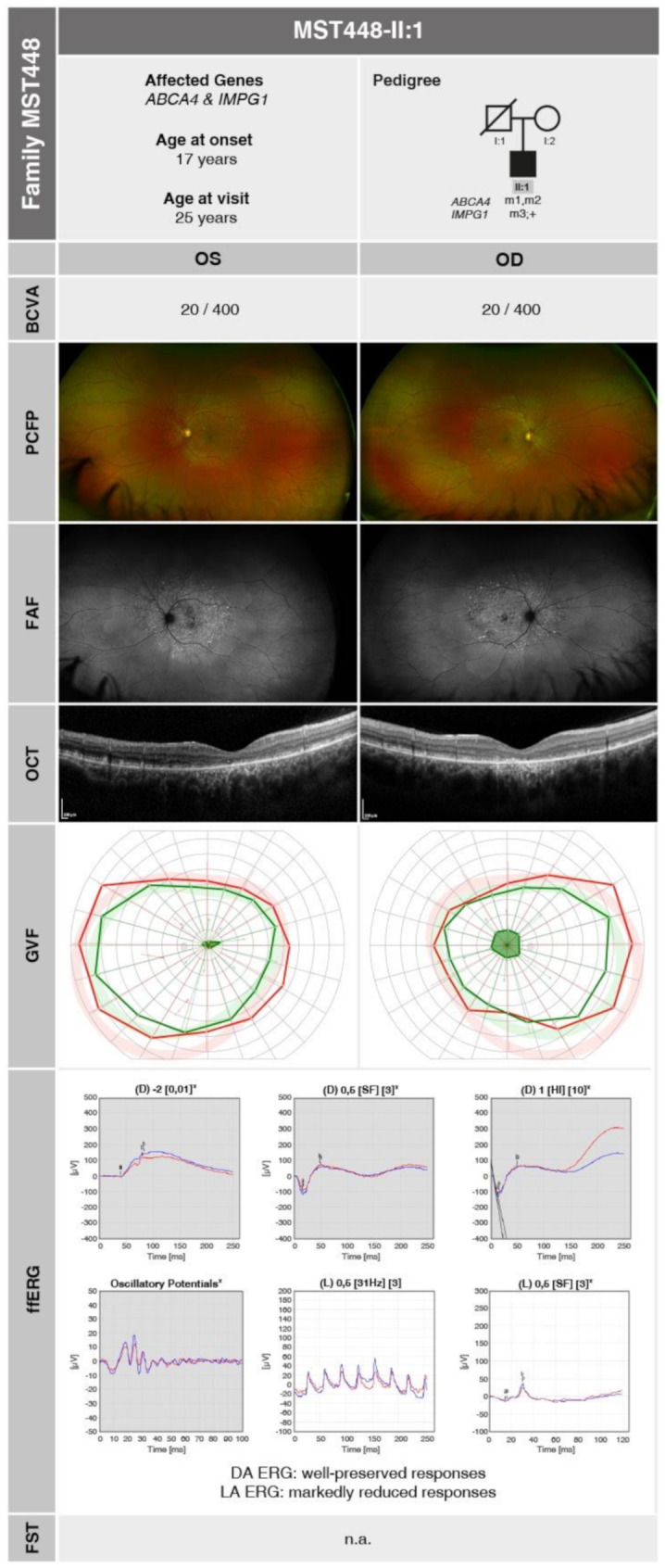
Pedigree analysis and summary of morphological and functional findings in **MST448-II:1** at the age of 25 years. The identified mutations are represented as follows: m1: *ABCA4* c.181 A > G, p.(Met61Val); m2: *ABCA4* c.3703 A > G, p.(Asn1235Asp); m3: *IMPG1* c.151dup, p.(Met51AsnfsTer29). The ffERG traces are presented with a grey background to indicate DA conditions and a white background to indicate LA conditions

He presented to our clinic at the age of 25 years, reporting progressive reduction in visual acuity since the age of 17 years and night blindness. At the time of consultation, his BCVA was reduced to 20/400 in both eyes (OD: -4.25 DS / -1.25 DC × 2°; OS: -3.50 DS / -1.00 DC × 4°). The anterior segment examination was unremarkable. Retinal examination and imaging revealed atrophy of the outer retinal layers and pigment epithelial changes in the central macula with corresponding hypoautofluorescence and the presence of flavimaculatus flecks at the posterior pole, characteristic features of STGD1. GVF demonstrated physiological outer boundaries with Goldmann size III4e and I4e stimuli, along with a central scotoma. ffERG showed reduced LA but well-preserved a-waves in DA responses with slightly reduced b-waves. A summary of genetic and clinical findings in MST448-II:1 is provided in Fig. 2.

### Family SRP1400: *ABCA4 & HK1*

The female patient SRP1400-II:1 was diagnosed with RP along with intellectual disability, associated with a *de novo* heterozygous likely pathogenic missense variant in the *HK1* gene (c.1334 C > T, p.(Ser445Leu)). Additionally, she carried the heterozygous pathogenic missense variant c.2588G > C, p.(Gly863Ala) (mild) in the *ACBA4* gene, which she inherited paternally and the heterozygous missense variant c.5642 C > T, p.(Ala1881Val) in the *ABCA4* gene which has non-uniform classification of likely pathogenic and variant of uncertain significance in ClinVar (https://www.ncbi.nlm.nih.gov/clinvar/), which she inherited from her mother.

**Fig. 3:**
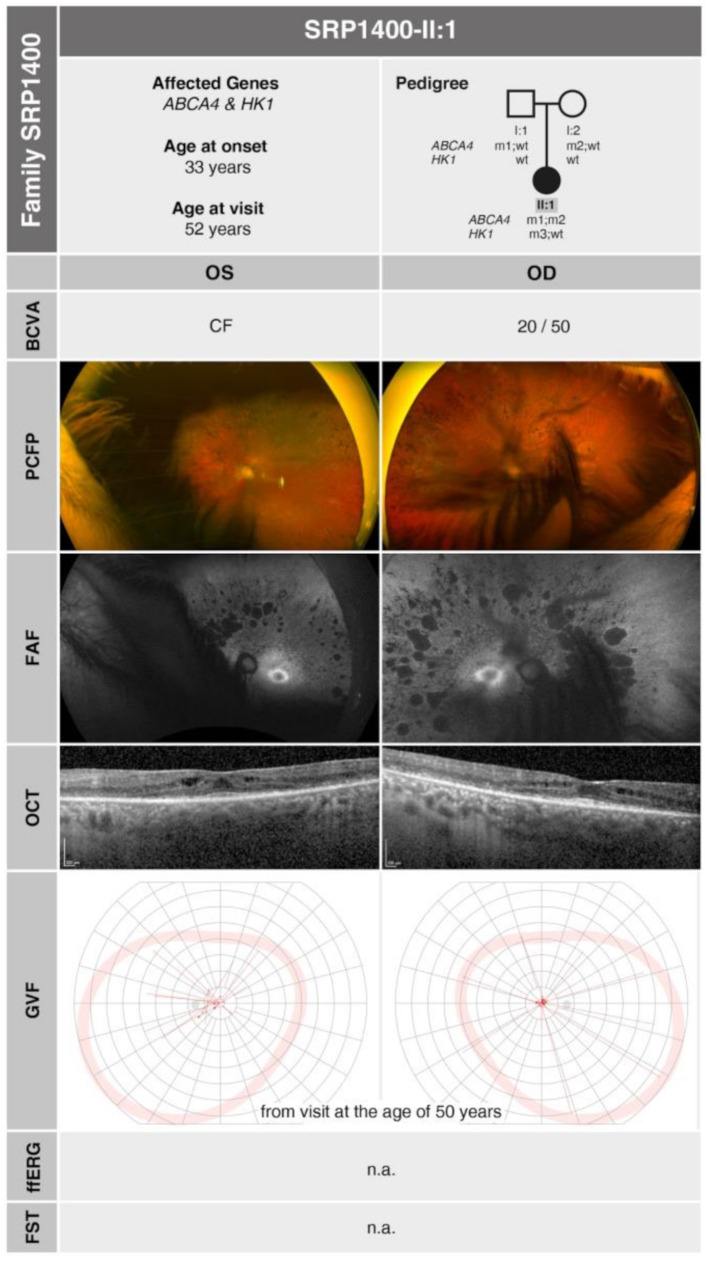
Pedigree analysis and summary of morphological and functional findings in **SRP1400-II:1** at the age of 52 years (GVF results are from the visit at the age of 50 years). The identified mutations are represented as follows: m1: *ABCA4* c.2588G > C, p.(Gly863Ala); m2: *ABCA4* c.5642 C > T, p.(Ala1881Val); m3: *HK1* c.1334 C > T, p.(Ser445Leu)

At the initial consultation at 37 years of age, she reported a gradual decline in visual acuity over the preceding 3–4 years. At that time, her BCVA was slightly reduced to 20/25 in the right eye (-1.00 DS) and 20/30 in the left eye (-1.00 DS / -1.00 DC × 95°). Examination of the anterior segment revealed incipient posterior subcapsular cataracts in both eyes. Retinal examination and imaging showed a waxy appearance of the optic disc and narrowed retinal vessels. Bone spicules and diffuse atrophies were observed predominantly in the mid-periphery, with a perifoveal hyperautofluorescent ring visible in both eyes in FAF. At a follow-up visit at age 41, her BCVA had further declined to 20/32 in the right eye and 20/40 in the left eye. GVF revealed concentric constriction of the visual field to approximately 3°. Fundoscopy showed multiple pigmentary changes extending to the center, consistent with a complete clinical picture of RP. Frequent follow-up visits in subsequent years revealed a progression of subcapsular and nuclear cataracts, along with a further reduction in visual acuity and visual field restriction. By the age of 52, her BCVA had decreased to 20/50 in the right eye and counting fingers (CF) in the left eye. OCT revealed diffuse outer retinal degeneration, with a small area of foveal sparing and mild perifoveal macular edema, typical for RP. Cataract surgery was indicated and performed, resulting in a post-operative BCVA of 20/50. A summary of genetic and clinical findings in SRP1400-II:1 is provided in Fig. 3.

### Family USHI105: *ABCA4 & MYO7A*

The male patient USHI105-II:1 was clinically diagnosed with RP associated with Usher syndrome type I. Genetic testing revealed a heterozygous pathogenic splicing variant (c.1555–8 C > G, p.(?)) and a heterozygous pathogenic missense variant (c.3503G > A, p.(Arg1168Gln)) in the *MYO7A* gene. Furthermore, he presented with three variants in *ABCA4*: the heterozygous deep intronic variant of uncertain significance c.4539 + 859 C > T, p.(?) (benign), the heterozygous common pathogenic splicing variant c.5461-10T > C, p.(?) (severe) as well as the homozygous missense variant c.5603 A > T, p.(Asn1868lle) (mild) that is classified as variant of uncertain significance, but is frequently associated with *ABCA4*-related IRD and considered a hypomorphic *ABCA4* allele. Segregation analysis in both parents confirmed the *MYO7A* variants to be biallelic and therefore causative for the Usher syndrome phenotype. Yet the genetic workup of the *ABCA4* variants was more complex. The father was shown to be a heterozygous carrier of the deep intronic variant c.4539 + 859 C > T and of the hypomorphic missense variant c.5603 A > T, p.(Asn1868lle). The mother is heterozygous for the pathogenic splicing variant c.5461-10T > C, p.(?) and is also homozygous for the hypomorphic missense variant c.5603 A > T, p.(Asn1868lle), thereby presenting with the very same *ABCA4* genotype as her son. This very genotype has been observed recurrently in patients with late onset STGD1 [32, 33].

**Fig. 4:**
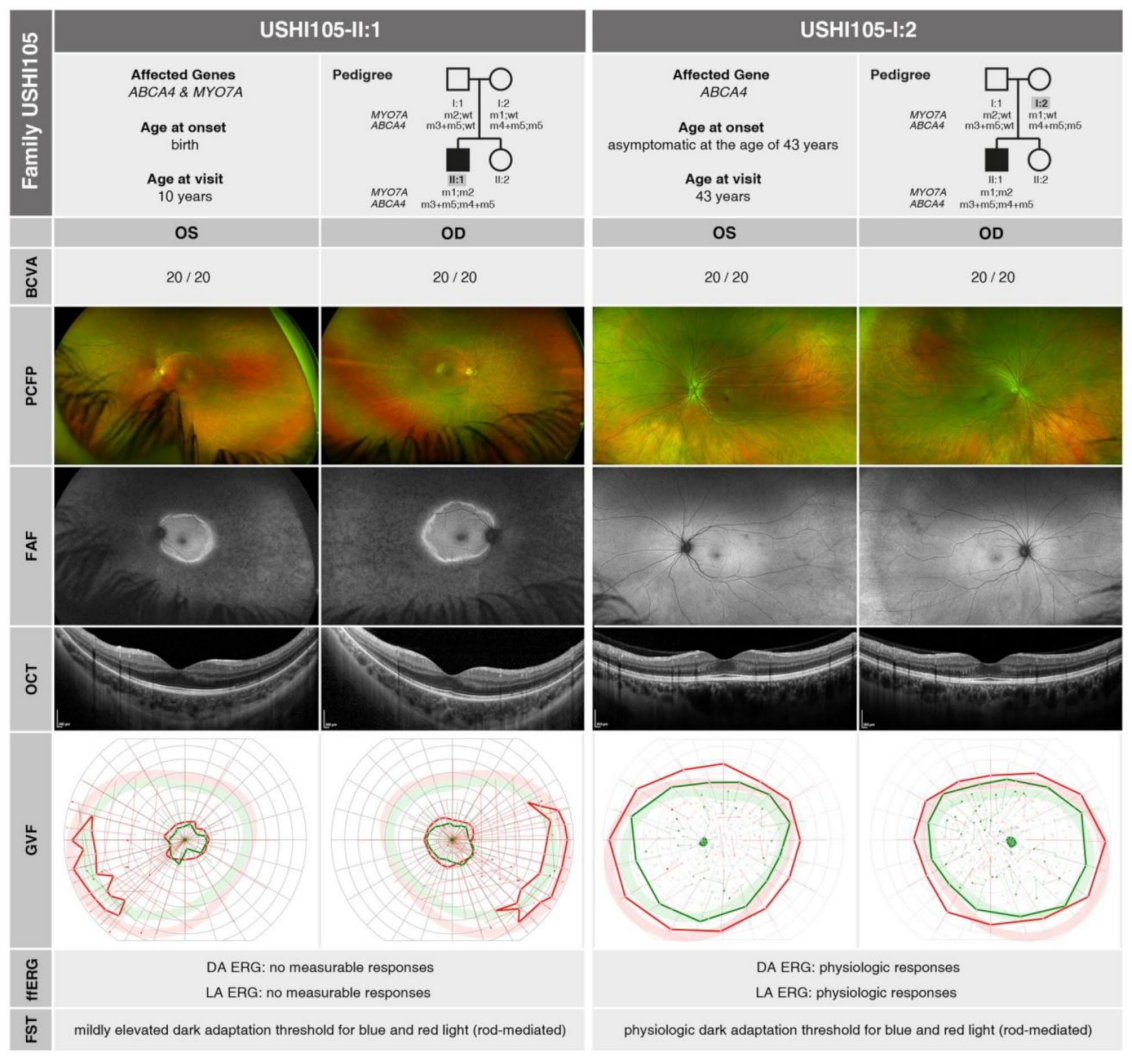
Pedigree analysis and summary of morphological and functional findings. The left panel shows **USHI105-II:1** at the age of 10 years and the right panel presents **USHI105-I:2** at the age of 43 years. The identified mutations are represented as follows: m1: *MYO7A* c.1555–8 C > G, p.(?); m2: *MYO7A* c.3503G > A, p.(Arg1168Gln); m3: *ABCA4* c.4539 + 859 C > T, p.(?); m4: *ABCA4* c.5461-10T > C, p.(?); m5: *ABCA4* c.5603 A > T, p.(Asn1868lle)

At the initial consultation at 10 years of age, USHI105-II:1 presented to our clinic with complaints of congenital deafness as well as a progressively narrowing visual field, night blindness and increased photophobia which had been worsening in the years leading up to the visit. BCVA testing revealed well-preserved visual acuity of 20/20 in both eyes (OD: -4.75 DS / -1.25 DC × 10°; OS: -5.25 DS / -0.75 DC × 180°). The anterior segment was unremarkable. Retinal examination and imaging showed attenuated vessels and diffuse atrophy outside the arcades with no bone spicules, while the macular region remained intact. FAF revealed a hyperautofluorescent ring along the arcades and a globally reduced autofluorescence pattern with hypoautofluorescent lesions outside the arcades. OCT showed atrophy of the outer retinal layers with extensive foveal sparing. GVF revealed concentric constriction to approximately 15–20° with temporal inferior islands using the Goldmann III4e stimulus. ffERG showed no measurable responses under both DA und LA conditions and FST indicated a rod-mediated, moderately elevated dark adaptation threshold for blue (-38.10 dB) and red (-17.83 dB) light. Overall, congenital deafness combined with the clinical presentation of RP suggested a suspected diagnosis of Usher syndrome type I-associated RP. A summary of genetic and clinical findings is provided in Fig. 4, **USHI105-II:1**.

As mentioned earlier, his mother, USHI105-I:2, is also homozygous for the *ABCA4* variant c.5603 A > T; p.(Asn1868Ile) and heterozygous for the *ABCA4* variant c.5461-10T > C, p.(?) and a heterozygous carrier of the *MYO7A* variant c.1555–8 C > G, p.(?). She presented to our clinic at the age of 43 years with no visual symptoms. At the time of consultation, her BCVA was fully preserved in both eyes (OD: -3.00 DS / -0.25 DC × 155°; OS: -2.75 DS / -1.50 DC × 15°). Both morphological and functional diagnostic assessments were unremarkable. Overall, USHI105-I:2 remains clinically asymptomatic, despite carrying the pathogenic heterozygous c.5461-10T > C, p.(?) variant and the hypomorphic homozygous c.5603 A > T, p.(Asn1868Ile) variant in *ABCA4*. A summary of genetic and clinical findings is provided in Fig. 4, **USHI105-I:2**.

## Discussion

We present four cases carrying a dual-gene variant constellation including biallelic pathogenic variants in the *ABCA4* gene. Additional variants in other retinal genes were assessed for their contribution to the different phenotypes. It is very difficult to define the impact of two potentially contributing genotypes, due to the complex interplay on the disease manifestation and progression. Additionally, it is well known that for *ABCA4*-associated IRDs the particular variants influence the severity of the retinal phenotype [34]. Hypomorphic alleles typically manifest in a milder disease, later onset or are even non-penetrant, whereas loss-of-function variants are more often associated with severe retinal dysfunction and degeneration.

In MST465-II:1, the presence of a dual-gene variant constellation with variants in both the *ABCA4* and *CACNA1F* genes creates a complex clinical picture with features clearly attributable to both gene-associated pathologies. The clinical presentation of MST465-II:1 exhibits hallmark features typical of *ABCA4*-associated retinal dystrophies, such as progressive atrophy of the outer retinal layers primarily affecting the central macula and flavimaculatus flecks. This aligns with a diagnosis of *ABCA4*-IRD, with early onset and a possible later progression into a cone-rod dystrophy, similar to the findings in his brother MST465-II:2 with a sole *ABCA4* genotype. Over a three-year follow-up period, MST465-II:1 experienced a severe reduction in visual acuity, highlighting the disease’s progressive nature. Beyond the *ABCA4*-related features, the variant in the *CACNA1F* gene introduces further clinical manifestations. Notably, MST465-II:1 exhibits nystagmus, a common feature in *CACNA1F*-associated disease. The presence of night blindness, typically unusual in young children with *ABCA4*-IRD, can also be attributed to the *CACNA1F* variant. This variant is known to cause iCSNB, a condition characterized by impaired night vision from birth and cone involvement [35, 36]. Electrophysiological findings further support the impact of the *CACNA1F* variant with markedly reduced responses not only under LA but also under DA conditions, which can also be seen in his brother MST465-II:3, carrying solely the pathogenic *CACNA1F* variant. In contrast, MST465-II:2, only carrying the disease-causing *ABCA4* genotype, shows reduced responses almost only under LA conditions in ffERG. All in all, MST465-II:1 presents a complex case of IRD, with clinical features attributable to variants in both the *ABCA4* and *CACNA1F* genes, with the single typical disease expressions manifesting in both his brothers, carrying the disease-causing genotype in either one of the genes. iCSNB is a stationary disorder, so any observed disease progression is unlikely due to the *CACNA1F* variant. While this variant influences the phenotype, the progressive nature of disease in MST465-II:1 remains primarily related to the *ABCA4*-associated pathology. Mechanistically, the *ABCA4* variants likely disrupt the clearance of toxic retinoid compounds and lead to their accumulation in photoreceptor cells [15], while the *CACNA1F* variant impairs synaptic transmission at the photoreceptor-bipolar cell junction due to calcium channel dysfunction [37]. Given the distinct subcellular localizations of these proteins, this mixed phenotype may rather arise from independent dysfunctions of ABCA4 and CACNA1F, rather than from a direct mechanistic interplay between the two malfunctioning proteins and is truly attributable to the dual-gene constellation.

In MST448-II:1, variants in both the *ABCA4* and *IMPG1* genes were considered potential contributors to the observed macular dystrophy. MST448-II:1 displays a phenotype consistent with STGD1, including fundus flavimaculatus, which is likely primarily related to the *ABCA4* variants. *IMPG1* plays a key role in maintaining the structural integrity and function of the interphotoreceptor matrix, which supports photoreceptor cells. However, MST448-II:1 does not exhibit the characteristic features of vitelliform macular dystrophy or RP typically associated with *IMPG1* variants [38], the latter supported by electrophysiological testing showing no rod involvement. In addition, evidence for the pathogenicity of the variant is ambiguous. The 1 bp duplication results in early frameshift and likely represents a null allele. It is also likely too common in the general population to represent a dominant acting variant (27 heterozygous entries, minor allele frequency 0.001675% in gnomAD v4.1.0 [39]). Variants in the *IMPG1* gene have been described related to both autosomal dominant and autosomal recessive inheritance [40, 41], but no clear disease mechanism and genotype-phenotype correlation has been achieved to date. It is plausible to assume that loss-of-function variants – as observed in our patient – are more likely to be recessive, while missense and small in-frame deletion or insertion variants may act dominant (negative). Therefore, while we cannot definitively confirm or exclude *IMPG1*’s contribution to the disease, the *IMPG1* variant does not seem to contribute to the phenotype in this patient, but rather should be considered a heterozygous carrier status. This shows that it is clinically challenging to differentiate between and assess the potential impact of such dual-gene variants on the IRD phenotype in any given patient and that retrospective critical re-evaluation of the phenotype depending on the identified genotype is essential.

In contrast to the first two cases, SRP1400-II:1 and USHI105-II:1 exhibit a phenotype more consistent with RP.

In SRP1400-II:1, the RP-like retinal degeneration and neurodevelopmental disorder appears to be primarily related to the presence of a *de novo HK1* variant, which is associated with autosomal dominant syndromal RP [42]. The *HK1* gene is involved in glycolysis and its disruption may lead to energy deficits in the highly metabolically active photoreceptors, particularly rods [42]. Pedigree and segregation analyses confirmed the *HK1* variant as *de novo* (Fig. 3). Clinically, SRP1400-II:1 exhibits a relatively typical RP phenotype, with progressive retinal degeneration noted during follow-up visits along with intellectual disability. While *ABCA4* variants have also been associated with RP (RP19), its primary disease spectrum includes cone-rod dystrophy, with RP-like features appearing in advanced cases [8]. They may contribute to the RP phenotype or have a subtle effect, potentially acting as modifiers rather than primary pathogenic drivers. ABCA4 and HK1 operate in distinct cellular pathways, ABCA4 in the visual cycle and HK1 in glucose metabolism. Due to the significant clinical variability of *HK1*-associated IRDs, which can manifest as RP, macular dystrophy or cone-rod dystrophy, even among individuals carrying the same variant [43], the precise contribution of *ABCA4* variants remains plausible but challenging to delineate with certainty.

In USHI105-II:1, the pathogenic biallelic *MYO7A* variants are the primary cause of the Usher-associated RP phenotype. *MYO7A* is crucial for retinal function, particularly within the retinal pigment epithelium, where it regulates melanosome distribution, phagosome transport and RPE65 translocation, essential for the visual cycle and photoreceptor maintenance [44]. Similar to SRP1400-II:1, the contribution of the described *ABCA4* variants is, however, not entirely clear. The patient is heterozygous for the c.5461-10T > C, p.(?) variant, which is pathogenic, and homozygous for the hypomorphic c.5603 A > T;p.(Asn1868Ile) variant. The latter is known for incomplete penetrance and only manifests a retinal phenotype when paired with a severe mutation on the counter-allele [32, 33, 45], and the c.5461-10T > C, p.(?) variant has been categorized as severe [7, 34]. Consequently, a contribution to the development of the IRD would be plausible as this very genotype has been reported to result in age-related macular degeneration (AMD)-like macular dystrophy and late onset STGD1 [32, 33]. It remains uncertain whether the irregular hyperautofluorescent ring around the macular region is an expression of an early stage of RP or the contribution of the *ABCA4* genotype to this less RP-typical formation of the hyperfluorescent ring. Although both MYO7A and ABCA4 are critical for retinal health, their roles in the eye involve distinct cellular processes. Given that Usher syndrome type 1 typically presents with early-onset, progressive RP [46], it is possible that *ABCA4*-related changes are masked or have not yet manifested. Segregation analysis shows that the mother, USHI105-I:2, is also homozygous for the c.5603 A > T; p.(Asn1868Ile) variant and heterozygous for the c.5461-10T > C, p.(?) variant in *ABCA4*, yet remains asymptomatic, consistent with the incomplete penetrance of the c.5603 A > T; p.(Asn1868Ile) variant and the late onset, probably characteristic for this very genotype [32, 33, 45].

In summary, MST465-II:1 exhibits a mixed phenotype clearly related to the combined pathophysiological effects of *ABCA4* and *CACNA1F* variants, while MST448-II:1 presents a typical case of STGD1, caused by the *ABCA4* genotype, while the contribution of the *IMPG1* variant is less likely. Conversely, SRP1400-II:1 and USHI105-II:1 display predominantly RP-like phenotypes, where the *ABCA4* genotype seem to play a more subtle role compared to the *HK1* or *MYO7A* variants, possibly functioning as modifiers rather than primary drivers of the disease at least at this time point. It is also possible that the phenotype exhibiting greater severity or more rapid onset tends to be more pronounced and conceal the phenotypic impact of the minor severe genotype.

This work highlights the importance of assessing the potential combined effects of multiple genetic variants and genotypes in patients with complex phenotypes, including extensive family segregation analysis, as these interactions can lead to overlapping or atypical clinical presentations. The intricate interplay between variants may produce features that are not easily attributed to a single genetic cause, as exemplified by the mixed phenotype observed in MST465-II:1, involving pathogenic variants in both the *ABCA4* and *CACNA1F* genes. Advanced diagnostic tools, such as multimodal clinical testing and exome or genome sequencing based “all IRD genes” panel testing, have been crucial in unraveling these genetic constellations. These methods not only identified the genetic factors contributing to the patients’ symptoms but also enhanced the overall diagnostic accuracy. Traditional panel analyses limited to a certain diagnostic entity (e.g. macular dystrophy only or RP only) might have missed pathogenic variants that do not align primarily with the expected phenotype. In particular, for MST465-II:1, this could have resulted in the *CACNA1F* variant being overlooked, as well as the detection of the mild iCSNB case in his younger brother MST465-II:3.

In the context of therapeutic approaches, it is necessary to mention that patients with dual-gene variant constellations might benefit less, or not at all, from potential future genetic therapies. In such cases, the potential therapeutic gain could be minimal, and the additional risks posed by the therapy, as well as the high costs involved, may not be justified. This highlights the importance of careful patient selection and complete genotype evaluation before initiating treatment.

Our study is not without limitations. Firstly, the sample size is small, primarily due to the rarity of dual-gene variant constellations. Within our large cohort recently published [5], we identified 12 such cases out of which these *ABCA4*-related cases were selected. Overlapping phenotypes like these observed in MST465-II:1 complicate the identification of the primary drivers of symptoms and the underlying morphological and functional abnormalities. From an investigational perspective, the differing genetical and clinical involvement among members MST465-II:1, MST465-II:2 and MST465-II:3 of the same family presents a unique opportunity to explore and dissect the individual contributions of dual-gene variants. However, it is important to acknowledge that such cases will likely remain rare and only described in small reports or individual cases. Longitudinal observations would further enrich our understanding of the molecular mechanisms, progression patterns and potential therapeutic strategies for these complex cases.

## Conclusions

In conclusion, this study highlights the complexity of IRDs associated with dual-gene variant constellations. The findings emphasize the necessity for advanced diagnostic techniques and thorough genotype evaluation to accurately unravel the contributions of multiple genetic factors. This approach is crucial for optimizing clinical decision-making and therapeutic strategies, ensuring that the benefits of potential future gene therapies outweigh their risks and costs.

## Data Availability

Raw data from whole genome sequencing are not publicly available to protect individuals’ privacy in compliance with the European General Data Protection Regulation (GDPR). Access to the data may be granted upon reasonable request and subject to appropriate ethical and legal approvals.

## Abbreviations

ACMG: American College of Medical Genetics and Genomics
BCVA: Best-corrected visual acuity
CF: Counting fingers
DA: Dark-adapted
FAF: Fundus autofluorescence
ffERG: Full-field electroretinography
FST: Full-field stimulus threshold
GVF: Goldmann visual field
iCSNB: Incomplete congenital stationary night blindness
IRD: Inherited retinal disease
LA: Light-adapted
OCT: Optical coherence tomography
PCFP: Pseudocolor fundus photography
RP: Retinitis pigmentosa
STGD1: Stargardt disease type 1

## References

[1] Villafuerte-de la Cruz RA, Garza-Garza LA, Garza-Leon M, Rodriguez-De la Torre C, Parra-Bernal C, Vazquez-Camas I et al. Spectrum of variants associated with inherited retinal dystrophies in Northeast Mexico. BMC Ophthalmol. 2024;24(1). 10.1186/s12886-023-03276-7 PMC10860328 38347443

[2] RetNet - Retinal Information Network [Internet]. Available from: https://retnet.org/.

[3] Daiger SP, Sullivan LS, Bowne SJ. Genes and mutations causing retinitis pigmentosa. Clin Genet. 2013;84(2):132–41. 23701314 10.1111/cge.12203 PMC3856531

[4] Stone EM, Andorf JL, Whitmore SS, DeLuca AP, Giacalone JC, Streb LM, et al. Clinically focused molecular investigation of 1000 consecutive families with inherited retinal disease. Ophthalmology. 2017;124(9):1314–31. 28559085 10.1016/j.ophtha.2017.04.008 PMC5565704

[5] Weisschuh N, Mazzola P, Zuleger T, Schaeferhoff K, Kühlewein L, Kortüm F, et al. Diagnostic genome sequencing improves diagnostic yield: a prospective single-centre study in 1000 patients with inherited eye diseases. J Med Genet. 2024;0:1–10. 10.1136/jmg-2023-109470 PMC10850689 37734845

[6] Nouri Z, Sarmadi A, Narrei S, Kianersi H, Kianersi F, Tabatabaiefar MA. Clinical characterizations and molecular genetic study of two co-segregating variants in PDZD7 and PDE6C genes leading simultaneously to non-syndromic hearing loss and achromatopsia. BMC Med Genomics. 2024;17(1). 10.1186/s12920-024-01942-3 PMC11218353 38956522

[7] Cornelis SS, Bauwens M, Haer-Wigman L, De Bruyne M, Pantrangi M, De Baere E, et al. Compendium of clinical variant classification for 2,246 unique ABCA4 variants to clarify variant pathogenicity in Stargardt disease using a modified ACMG/AMP framework. Hum Mutat. 2023;2023(1):6815504. 40225145 10.1155/2023/6815504 PMC11918811

[8] Cremers FPM, Lee W, Collin RWJ, Allikmets R. Clinical spectrum, genetic complexity and therapeutic approaches for retinal disease caused by ABCA4 mutations. Prog Retin Eye Res. 2020;79:100861. 32278709 10.1016/j.preteyeres.2020.100861 PMC7544654

[9] Watson A, Queen R, Ferrández-Peral L, Dorgau B, Collin J, Nelson A, et al. Unravelling genotype-phenotype correlations in Stargardt disease using patient-derived retinal organoids. Cell Death Dis. 2025;16(1):1–15. 39971915 10.1038/s41419-025-07420-7 PMC11840025

[10] Cideciyan AV, Swider M, Aleman TS, Tsybovsky Y, Schwartz SB, Windsor EAM, et al. ABCA4 disease progression and a proposed strategy for gene therapy. Hum Mol Genet. 2009;18(5):931–41. 19074458 10.1093/hmg/ddn421 PMC2640207

[11] Rozet JM, Gerber S, Ghazi I, Perrault I, Ducroq D, Souied E, et al. Mutations of the retinal specific ATP binding transporter gene (ABCR) in a single family segregating both autosomal recessive retinitis pigmentosa RP19 and Stargardt disease: evidence of clinical heterogeneity at this locus. J Med Genet. 1999;36(6):447–51. 10874631 PMC1734380

[12] Tanna P, Strauss RW, Fujinami K, Michaelides M. Stargardt disease: clinical features, molecular genetics, animal models and therapeutic options. Br J Ophthalmol. 2017;101(1):25–30. 27491360 10.1136/bjophthalmol-2016-308823 PMC5256119

[13] Al-khuzaei S, Broadgate S, Foster CR, Shah M, Yu J, Downes SM, et al. An overview of the genetics of ABCA4 retinopathies, an evolving story. Genes 2021. 2021;12(8):1241. 12 Page 1241. 10.3390/genes12081241 PMC8392661 34440414

[14] Cornelis SS, Runhart EH, Bauwens M, Corradi Z, De Baere E, Roosing S, et al. Personalized genetic counseling for Stargardt disease: offspring risk estimates based on variant severity. Am J Hum Genet. 2022;109(3):498–507. 35120629 10.1016/j.ajhg.2022.01.008 PMC8948157

[15] Molday RS, Garces FA, Scortecci JF, Molday LL. Structure and function of ABCA4 and its role in the visual cycle and Stargardt macular degeneration. Prog Retin Eye Res. 2022;89:101036. 34954332 10.1016/j.preteyeres.2021.101036

[16] Huynh N, Jeffrey BG, Turriff A, Sieving PA, Cukras CA. Sorting out Co-occurrence of rare Monogenic retinopathies: Stargardt disease Co-existing with congenital stationary night blindness. Ophthalmic Genet. 2014;35(1):51–6. 24397708 10.3109/13816810.2013.865762 PMC8325388

[17] Lee W, Schuerch K, Xie Y, Zernant J, Tsang SH, Sparrow JR, et al. Simultaneous expression of ABCA4 and GPR143 mutations: A complex phenotypic manifestation. Invest Ophthalmol Vis Sci. 2016;57(7):3409–15. 27367509 10.1167/iovs.16-19621 PMC4961055

[18] Hayashi T, Hosono K, Kurata K, Katagiri S, Mizobuchi K, Ueno S, et al. Coexistence of GNAT1 and ABCA4 variants associated with Nougaret-type congenital stationary night blindness and childhood-onset cone-rod dystrophy. Doc Ophthalmol. 2020;140(2):147–57. 31583501 10.1007/s10633-019-09727-1

[19] Stevanovic M, O’Connell K, Gupta P, Borchert JS, Comander J, Place E, et al. Dual molecular diagnoses in patients with inherited retinal degenerations. Invest Ophthalmol Vis Sci. 2023;64(8):4635.

[20] Tan TY, Lunke S, Chong B, Phelan D, Fanjul-Fernandez M, Marum JE, et al. A head-to-head evaluation of the diagnostic efficacy and costs of trio versus Singleton exome sequencing analysis. Eur J Hum Genet. 2019;27(12):1791–9. 31320747 10.1038/s41431-019-0471-9 PMC6871178

[21] Richards S, Aziz N, Bale S, Bick D, Das S, Gastier-Foster J, et al. Standards and guidelines for the interpretation of sequence variants: a joint consensus recommendation of the American college of medical genetics and genomics and the association for molecular pathology. Genet Med. 2015;17(5):405–24. 25741868 10.1038/gim.2015.30 PMC4544753

[22] Maugeri A, Klevering BJ, Rohrschneider K, Blankenagel A, Brunner HG, Deutman AF, et al. Mutations in the ABCA4 (ABCR) gene are the major cause of autosomal recessive Cone-Rod dystrophy. Am J Hum Genet. 2000;67(4):960–6. 10958761 10.1086/303079 PMC1287897

[23] Strom TM, Nyakatura G, Apfelstedt-Sylla E, Hellebrand H, Lorenz B, Weber BH, et al. An L-type calcium-channel gene mutated in incomplete X-linked congenital stationary night blindness. Nat Genet. 1998;19(3):260–3. 9662399 10.1038/940

[24] Jaakson K, Zernant J, Külm M, Hutchinson A, Tonisson N, Glavač D, et al. Genotyping microarray (Gene Chip) for the ABCR (ABCA4) gene. Hum Mutat. 2003;22(5):395–403. 14517951 10.1002/humu.10263

[25] Allikmets R, Singh N, Sun H, Shroyer NF, Hutchinson A, Chidambaram A, et al. A photoreceptor cell-specific ATP-binding transporter gene (ABCR) is mutated in recessive Starqardt macular dystrophy. Nat Genet 1997 153. 1997;15(3):236–46. 10.1038/ng0397-236 9054934

[26] Testa F, Rossi S, Sodi A, Passerini I, Di Iorio V, Corte MD, et al. Correlation between photoreceptor layer integrity and visual function in patients with Stargardt disease: implications for gene therapy. Invest Ophthalmol Vis Sci. 2012;53(8):4409–15. 22661472 10.1167/iovs.11-8201 PMC4625823

[27] Okur V, Cho MT, van Wijk R, van Oirschot B, Picker J, Coury SA, et al. De Novo variants in HK1 associated with neurodevelopmental abnormalities and visual impairment. Eur J Hum Genet EJHG. 2019;27(7):1081–9. 30778173 10.1038/s41431-019-0366-9 PMC6777464

[28] Klevering BJ, Deutman AF, Maugeri A, Cremers FPM, Hoyng CB. The spectrum of retinal phenotypes caused by mutations in the ABCA4 gene. Graefes Arch Clin Exp Ophthalmol Albrecht Von Graefes Arch Klin Exp Ophthalmol. 2005;243(2):90–100. 10.1007/s00417-004-1079-4 15614537

[29] Webster AR, Héon E, Lotery AJ, Vandenburgh K, Casavant TL, Oh KT, et al. An analysis of allelic variation in the ABCA4 gene. Invest Ophthalmol Vis Sci. 2001;42(6):1179–89. 11328725

[30] Weston MD, Kelley PM, Overbeck LD, Wagenaar M, Orten DJ, Hasson T, et al. Myosin VIIA mutation screening in 189 Usher syndrome type 1 patients. Am J Hum Genet. 1996;59(5):1074–83. 8900236 PMC1914835

[31] Aparisi MJ, Aller E, Fuster-García C, García-García G, Rodrigo R, Vázquez-Manrique RP, et al. Targeted next generation sequencing for molecular diagnosis of Usher syndrome. Orphanet J Rare Dis. 2014;9:168. 25404053 10.1186/s13023-014-0168-7 PMC4245769

[32] Zernant J, Lee W, Collison FT, Fishman GA, Sergeev YV, Schuerch K, et al. Frequent hypomorphic alleles account for a significant fraction of ABCA4 disease and distinguish it from age-related macular degeneration. J Med Genet. 2017;54(6):404–12. 28446513 10.1136/jmedgenet-2017-104540 PMC5786429

[33] Li CHZ, Pas JAAH, Corradi Z, Hitti-Malin RJ, Hoogstede A, Runhart EH, et al. Study of Late-Onset Stargardt type 1 disease characteristics, genetics, and progression. Ophthalmology. 2024;131:87–97. 37598860 10.1016/j.ophtha.2023.08.011

[34] Lee W, Zernant J, Su PY, Nagasaki T, Tsang SH, Allikmets R. A genotype-phenotype correlation matrix for ABCA4 disease based on long-term prognostic outcomes. JCI Insight. 2022;7(2). 10.1172/jci.insight.156154 PMC8855796 34874912

[35] Boycott KM, Maybaum TA, Naylor MJ, Weleber RG, Robitaille J, Miyake Y, et al. A summary of 20 CACNA1F mutations identified in 36 families with incomplete X-linked congenital stationary night blindness, and characterization of splice variants. Hum Genet. 2001;108(2):91–7. 11281458 10.1007/s004390100461

[36] Lambertus S, Van Huet RAC, Bax NM, Hoefsloot LH, Cremers FPM, Boon CJF, et al. Early-Onset Stargardt disease phenotypic and genotypic characteristics. Ophthalmology. 2015;122:335–44. 25444351 10.1016/j.ophtha.2014.08.032

[37] Mansergh F, Orton NC, Vessey JP, Lalonde MR, Stell WK, Tremblay F, et al. Mutation of the calcium channel gene Cacna1f disrupts calcium signaling, synaptic transmission and cellular organization in mouse retina. Hum Mol Genet. 2005;14(20):3035–46. 16155113 10.1093/hmg/ddi336

[38] Ishikawa M, Sawada Y, Yoshitomi T. Structure and function of the interphotoreceptor matrix surrounding retinal photoreceptor cells. Exp Eye Res. 2015;133:3–18. 25819450 10.1016/j.exer.2015.02.017

[39] Chen S, Francioli LC, Goodrich JK, Collins RL, Kanai M, Wang Q, et al. A genomic mutational constraint map using variation in 76,156 human genomes. Nature. 2024;625(7993):92–100. 38057664 10.1038/s41586-023-06045-0 PMC11629659

[40] Manes G, Meunier I, Avila-Fernández A, Banfi S, Le Meur G, Zanlonghi X, et al. Mutations in IMPG1 cause vitelliform macular dystrophies. Am J Hum Genet. 2013;93(3):571–8. 23993198 10.1016/j.ajhg.2013.07.018 PMC3769927

[41] Olivier G, Corton M, Intartaglia D, Verbakel SK, Sergouniotis PI, Le Meur G, et al. Pathogenic variants in IMPG1 cause autosomal dominant and autosomal recessive retinitis pigmentosa. J Med Genet. 2021;58(8):570–8. 32817297 10.1136/jmedgenet-2020-107150

[42] Sullivan LS, Koboldt DC, Bowne SJ, Lang S, Blanton SH, Cadena E, et al. A dominant mutation in hexokinase 1 (HK1) causes retinitis pigmentosa. Invest Ophthalmol Vis Sci. 2014;55(11):7147–58. 25190649 10.1167/iovs.14-15419 PMC4224580

[43] Yuan Z, Li B, Xu M, Chang EY, Li H, Yang L, et al. The phenotypic variability of HK1-associated retinal dystrophy. Sci Rep. 2017;7(1):7051. 28765615 10.1038/s41598-017-07629-3 PMC5539152

[44] Williams DS, Lopes VS. The many different cellular functions of MYO7A in the retina. Biochem Soc Trans. 2011;39(5):1207–10. 21936790 10.1042/BST0391207 PMC3703834

[45] Runhart EH, Sangermano R, Cornelis SS, Verheij JBGM, Plomp AS, Boon CJF, et al. The common ABCA4 variant p.Asn1868Ile shows nonpenetrance and variable expression of Stargardt disease when present in trans with severe variants. Invest Ophthalmol Vis Sci. 2018;59(8):3220–31. 29971439 10.1167/iovs.18-23881

[46] Weil D, Blanchard S, Kaplan J, Guilford P, Gibson F, Walsh J, et al. Defective myosin VIIA gene responsible for Usher syndrome type IB. Nat 1995 3746517. 1995;374(6517):60–1. 10.1038/374060a0 7870171

